# Deletion of CDKAL1 Affects Mitochondrial ATP Generation and First-Phase Insulin Exocytosis

**DOI:** 10.1371/journal.pone.0015553

**Published:** 2010-12-09

**Authors:** Mica Ohara-Imaizumi, Masashi Yoshida, Kyota Aoyagi, Taro Saito, Tadashi Okamura, Hitoshi Takenaka, Yoshihiro Akimoto, Yoko Nakamichi, Rieko Takanashi-Yanobu, Chiyono Nishiwaki, Hayato Kawakami, Norihiro Kato, Shin-ichi Hisanaga, Masafumi Kakei, Shinya Nagamatsu

**Affiliations:** 1 Department of Biochemistry, Kyorin University School of Medicine, Tokyo, Japan; 2 First Department of Medicine, Saitama Medical Center, Jichi Medical University School of Medicine, Saitama, Japan; 3 Department of Biological Sciences, Tokyo Metropolitan University, Tokyo, Japan; 4 Department of Infectious Diseases, Research Institute, National Center for Global Health and Medicine, Tokyo, Japan; 5 Department of Anatomy, Kyorin University School of Medicine, Tokyo, Japan; 6 Department of Gene Diagnostics and Therapeutics, Research Institute, National Center for Global Health and Medicine, Tokyo, Japan; VTT Technical Research Centre of Finland, Finland

## Abstract

**Background:**

A variant of the *CDKAL1* gene was reported to be associated with type 2 diabetes and reduced insulin release in humans; however, the role of CDKAL1 in β cells is largely unknown. Therefore, to determine the role of CDKAL1 in insulin release from β cells, we studied insulin release profiles in *CDKAL1* gene knockout (*CDKAL1* KO) mice.

**Principal Findings:**

Total internal reflection fluorescence imaging of *CDKAL1* KO β cells showed that the number of fusion events during first-phase insulin release was reduced. However, there was no significant difference in the number of fusion events during second-phase release or high K^+^-induced release between WT and KO cells. CDKAL1 deletion resulted in a delayed and slow increase in cytosolic free Ca^2+^ concentration during high glucose stimulation. Patch-clamp experiments revealed that the responsiveness of ATP-sensitive K^+^ (K_ATP_) channels to glucose was blunted in KO cells. In addition, glucose-induced ATP generation was impaired. Although CDKAL1 is homologous to cyclin-dependent kinase 5 (CDK5) regulatory subunit-associated protein 1, there was no difference in the kinase activity of CDK5 between WT and *CDKAL1* KO islets.

**Conclusions/Significance:**

We provide the first report describing the function of CDKAL1 in β cells. Our results indicate that CDKAL1 controls first-phase insulin exocytosis in β cells by facilitating ATP generation, K_ATP_ channel responsiveness and the subsequent activity of Ca^2+^ channels through pathways other than CDK5-mediated regulation.

## Introduction

Type 2 diabetes is a multifactorial disease characterized by decreased insulin secretion and decreased insulin action at target tissues. While the primary molecular defects in type 2 diabetes remain largely unknown, genetic factors in combination with environmental factors are thought to be involved in the onset and development of the disease. Recent genome-wide association studies have identified *CDKAL1* (cyclin-dependent kinase 5 regulatory subunit associated protein 1-like 1) as a susceptibility gene for type 2 diabetes, which has been replicated in several populations [1]–[5]. The *CDKAL1* gene is located on chromosome 6p22.3 and encodes a 65-kDa protein (CDKAL1). The expression of *CDKAL1* mRNA has been detected in human tissues including pancreatic islets, skeletal muscle and brain [1], [4]. Although the function of CDKAL1 is still unclear, CDKAL1 is similar to cyclin-dependent kinase 5 regulatory subunit-associated protein 1 (CDK5RAP1), which is expressed in neuronal tissues, and inhibits cyclin-dependent kinase 5 (CDK5) activity by binding to the CDK5 activator p35 [6]. CDK5 was previously implicated in islet function [7]–[9], suggesting that CDKAL1 plays a role in β cell function by inhibiting CDK5 kinase activity [1]–[4].

Genome-wide association studies have shown that several single-nucleotide polymorphisms (SNPs) in intron 5 of the *CDKAL1* gene are associated with type 2 diabetes [1]–[4]. An association between SNPs and insulin release has also been suggested [2], [3]. Recently, Groenewoud et al. [10] and Stancakova et al. [11] reported that a *CDKAL1* variant (rs7754840) decreased first-phase insulin secretion but not second-phase insulin secretion during hyperglycemic clamps and intravenous glucose tolerance tests, respectively. Furthermore, the gene variants were not associated with the insulin sensitivity index [10], [11], suggesting that *CDKAL1* variants influence the risk of type 2 diabetes by impairing first-phase insulin secretion. However, the molecular mechanisms through which CDKAL1 modulates insulin release in pancreatic β cells are unknown.

To understand the role of CDKAL1 in β cells, we studied the phenotype of mice deficient in CDKAL1 expression, which was established using a gene-trap technique (*CDKAL1* KO mice). Our data demonstrate that CDKAL1 has a role in first-phase insulin exocytosis in β cells by facilitating ATP generation, K_ATP_ channel responsiveness and subsequent Ca^2+^ channel activity; surprisingly, these effects were independent of CDK5 activity.

## Results

### Expression and histological examination of *CDKAL1* KO mice

**Figure 1:**
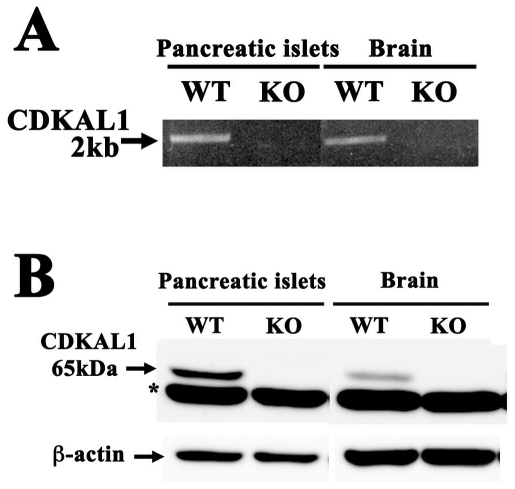
CDKAL1 expression is absent in *CDKAL1* KO mice. **A**. RT-PCR analysis of pancreatic islets and the whole brain of WT and *CDKAL1* KO mice. The *CDKAL1* transcript was not detected in *CDKAL1* KO mice. **B**. Immunoblot analysis. Homogenates of mouse pancreatic islets and whole brain (30 µg) were subjected to SDS-PAGE and immunoblotted with anti-CDKAL1 antibody. The protein band below the CDKAL1 protein (*) is a nonspecific protein band detected by the anti-CDKAL1 antibody.

**Figure 2:**
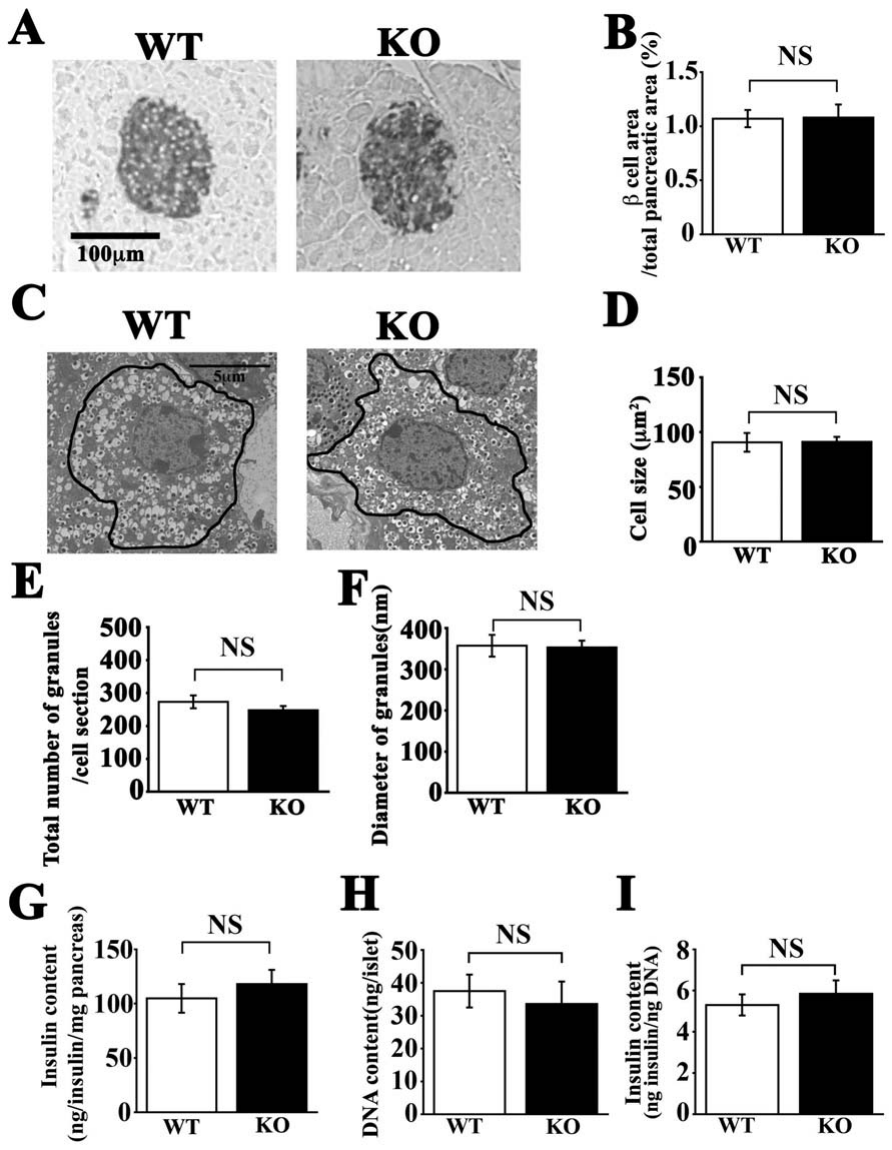
Pancreatic histology and insulin content in *CDKAL1* KO mice. **A**. *CDKAL1* KO mice have normal islet architecture. Pancreatic sections were peroxidase stained for insulin. Scale bar: 100 µm. **B**. Relative area occupied by β cells (percentage of total pancreatic area). Random sections of the entire pancreas from WT and *CDKAL1* KO mice were immunostained (as shown in **A**) and analyzed (60 sections from each of three mice per group). **C–F**. Electron micrographs of pancreatic tissue sections. **C** Representative sections (scale bar: 5 µm), **D** β cell size, **E** total number of granules per cell section, and **F** mean granule diameter in ultra-thin sections (100 nm) (n = 20 cells per group) of *CDKAL1* KO and WT β cells. **G–I**. Insulin content in *CDKAL1* KO mice. **G** Pancreatic insulin content measured in acid-ethanol extracts from WT and KO mice by ELISA (n = 6 per group). **H** DNA content per islet and **I** islet insulin content per DNA from WT and KO mice (n = 6 per group). Results are means±SEM.

To explore the potential role of CDKAL1 in β cells, we studied *CDKAL1* KO β cells. *CDKAL1* KO mice did not express *CDKAL1* mRNA in pancreatic islets or the brain (Fig. 1A). In immunoblot analysis (Fig. 1B), CDKAL1 protein was not detected in KO islets or KO brain, whereas it was more highly expressed in WT islets than in WT brain. We next morphologically examined the pancreatic islets. Immunostaining of pancreatic sections with insulin antibodies revealed no significant difference in islet architecture, including β cell area per pancreas, between WT and KO mice (Fig. 2A–B). Electron microscopy of the pancreatic β cells also revealed that cell size, total number of granules per section and mean granule diameter were similar in WT and KO β cells (Fig. 2C–F). Furthermore, pancreatic insulin content, islet DNA content and islet insulin content of *CDKAL1* KO mice were similar to those of WT mice (Fig. 2G–I). Thus, *CDKAL1* KO β cells displayed specific CDKAL1 protein depletion but their characteristics were similar to those of WT cells.

### Effects of *CDKAL1* KO on glucose-induced biphasic insulin exocytosis

**Figure 3:**
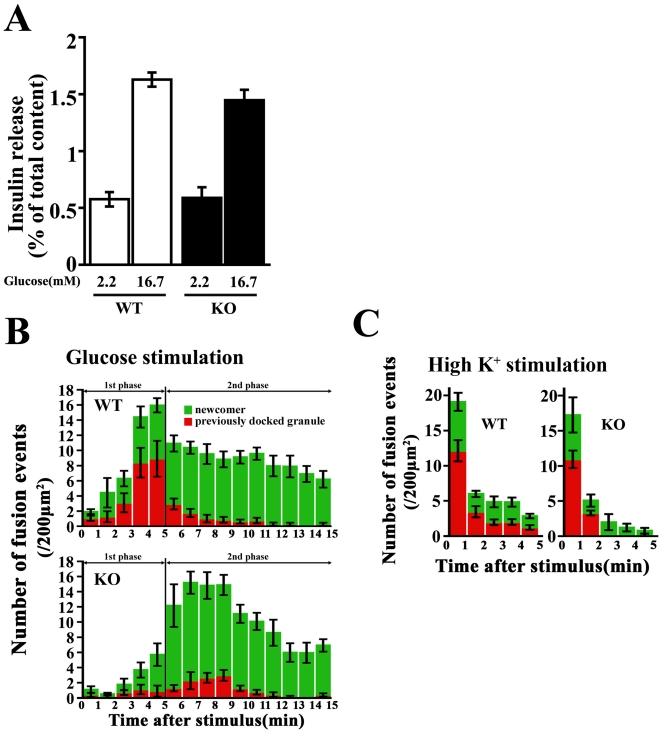
Effects of *CDKAL1* KO on glucose-induced biphasic insulin exocytosis. **A**. Insulin release (for 30 min) in batch-incubated WT and *CDKAL1* KO β cells in the presence of 2.2 mM or 16.7 mM glucose (n = 8 per group). **B**. Histogram showing the number of fusion events from GFP-tagged granules in wild-type (WT) and *CDKAL1* KO β cells (per 200 µm^2^) at 1-min intervals after stimulation with 22 mM glucose and measured by TIRF microscopy. Data are mean±SEM (WT, n = 16 cells; KO, n = 14 cells). Time 0 indicates the addition of 22 mM glucose. The red column shows fusion events from previously docked granules, and the green column shows those from newcomers. **C**. Histogram showing the number of fusion events in WT and KO β cells (per 200 µm^2^) at 1-min intervals after 40 mM high K^+^ stimulation measured by TIRF microscopy (n = 8 cells per group).

We next investigated the effects of *CDKAL1* KO on glucose-induced insulin release from β cells. Batch experiments showed that 16.7 mM glucose-induced insulin release (for 30 min) from *CDKAL1* KO β cells was decreased (∼19%) relative to that from WT β cells (*P* = NS) (Fig. 3A). To evaluate in detail the effects of CDKAL1 deficiency on glucose-evoked biphasic insulin exocytosis, the dynamic motion of individual insulin granules tagged with green fluorescent protein (GFP) was observed under total internal reflection fluorescence (TIRF) microscopy. As previously reported [12], stimulation with 22 mM glucose evoked biphasic insulin granule exocytosis through fusion of two types of granules (previously docked granules and newcomers) in WT β cells (Fig. 3B, top). During the first phase (within 5 min), the fusion events mostly occurred from the previously docked granules with some of the fusion events from newcomers while continuous fusion was observed thereafter, mostly from newcomers during the second phase (over 15 min). In contrast, *CDKAL1* KO β cells showed abnormalities in biphasic insulin granule exocytosis (Fig. 3B, bottom) because the number of fusion events during the first phase was significantly reduced in KO cells [WT (n = 16) vs. KO (n = 14), 43.0±6.2 vs. 12.7±3.8, P<0.001]. In contrast, during the second phase, the number of fusion events tended to be increased but there was no significant difference [WT (n = 16) vs. KO (n = 14), 87.3±6.4 vs. 105.6±8.0, P = NS].

**Figure 4:**
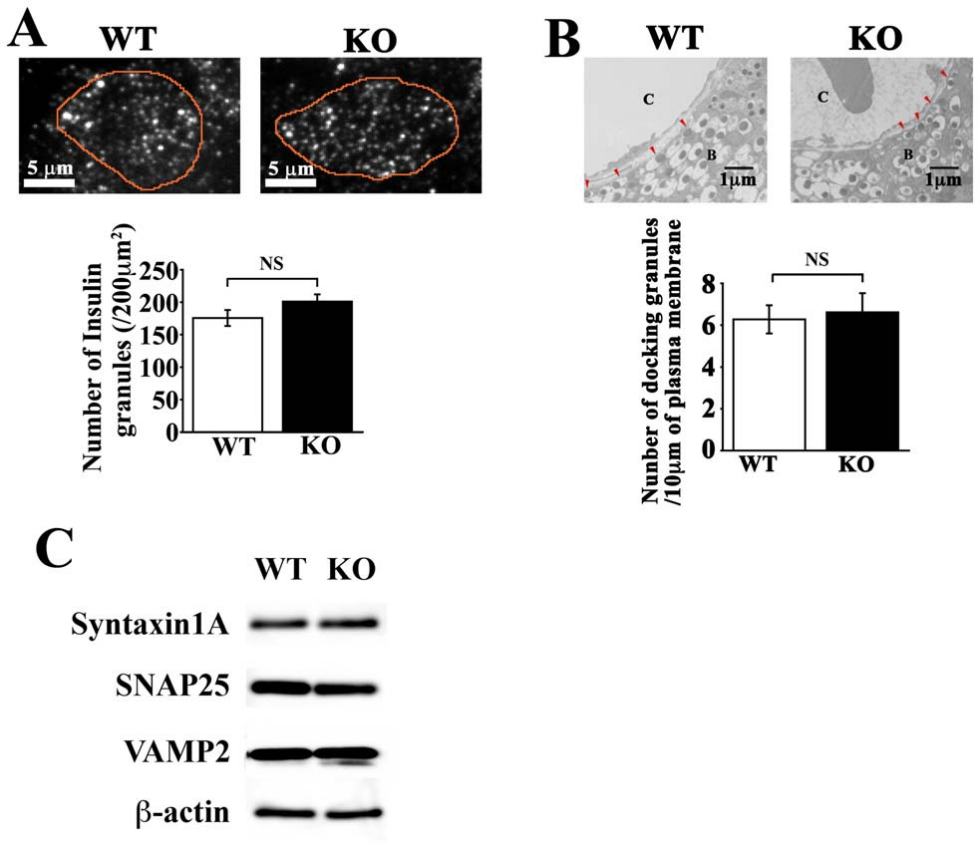
*CDKAL1* KO did not affect the number of morphologically docked granules or granule fusion kinetics. **A**. Total internal reflection fluorescence (TIRF) microscopy of insulin granules morphologically docked to the plasma membrane. (top) Typical TIRF images of docked insulin granules in WT and *CDKAL1* KO β cells. The surrounding lines represent the outline of cells attached to the cover glass. Scale bar: 5 µm. Pancreatic β cells were prepared from WT and KO mice, fixed, and immunostained for insulin. (bottom) Number of insulin granules morphologically docked to the plasma membrane. Individual fluorescent spots shown in the TIRF images were manually counted per 200 µm^2^ in 15 cells per group. **B**. Electron micrograph of β cell sections. (top) Typical images of the plasma membrane area facing the blood capillary (C) of WT and KO β cells (B). Bar: 500 nm. (bottom) Number of morphologically docked insulin granules per 10 µm of the plasma membrane. Granules at their shortest distance of <10 nm from the plasma membrane were defined as morphologically docked granules (red arrowheads). Results are means±SEM. **C**. Expression of SNARE proteins in wild-type (WT) and KO islets by immunoblotting. Equal amounts of islet protein (30 µg) were separated by SDS-PAGE and immunoblotted. β-actin was used as a loading control.

On the other hand, high K^+^-induced granule fusion was normal in KO cells (Fig. 3C), suggesting that the exocytotic steps after the elevation of intracellular calcium concentration ([Ca^2+^]_i_) were not impaired in KO cells. In fact, the number of morphologically docked insulin granules observed by TIRF and electron microscopy (Fig. 4A and B), and fusion kinetics (data not shown) were not altered in *CDKAL1* KO β cells. In addition, the expression levels of SNARE proteins (syntaxin1A, SNAP25 and VAMP2), important mediators of insulin granule docking and fusion [12], were similar in WT and KO β cells (Fig. 4C). These results indicate that granule docking and Ca^2+^-triggered fusion are not impaired in *CDKAL1* KO β cells.

### Changes in the [Ca^2+^]_i_ increase in KO cells

**Figure 5:**
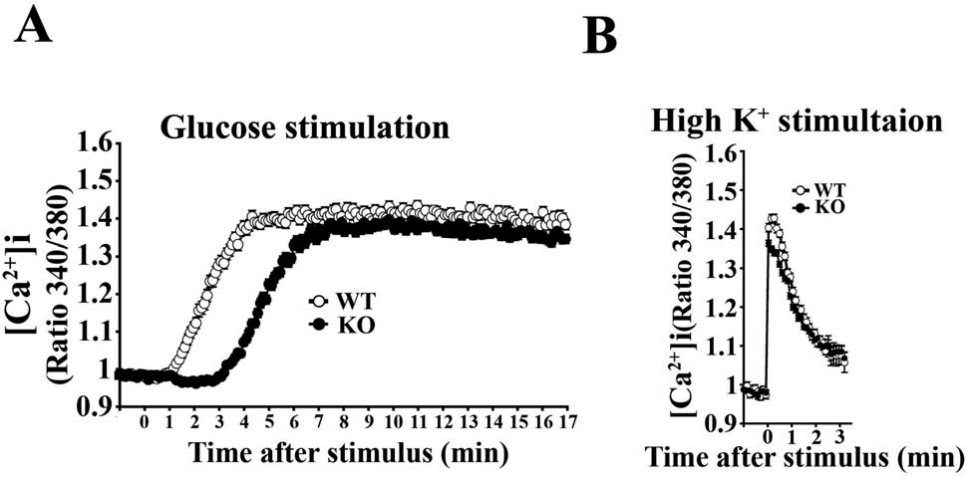
Effects of *CDKAL1* KO on changes in intracellular free calcium ([Ca^2+^]_i_). **A** 22 mM glucose- and **B** 40 mM high K^+^-induced changes in [Ca^2+^]_i_ in WT and *CDKAL1* KO β cells. Changes in [Ca^2+^]_i_ were measured by Fura-2 acetoxymethyl (2 µM). Time 0 indicates when the stimulants were added. The fluorescence ratio (340/360) at time 0 was taken as 1. Results are means±SEM (n = 12 cells per group).

To determine whether the impairments in glucose-induced first-phase insulin exocytosis were associated with changes in the [Ca^2+^]_i_ increase, we monitored the time-course of the glucose-induced [Ca^2+^]_i_ increase using Fura-2 methods. *CDKAL1* KO resulted in a delayed and slow increase in [Ca^2+^]_i_ during high glucose stimulation with a similar time-course to insulin exocytosis in KO cells. In contrast, a rapid and marked [Ca^2+^]_i_ rise was observed in WT cells (Fig. 5A). The mean lag time (Δ time from stimulation to onset) and rise time (Δ time from onset to peak) were slower in KO cells than in WT cells [WT vs. KO (n = 12 each) lag time: 1.2±0.1 min vs. 3.0±0.1 min, P<0.0001; rise time: 3.0±0.2 min vs. 4.1±0.2 min, P<0.001]. The magnitude of the [Ca^2+^]_i_ rise was not different between KO and WT cells. Furthermore, there was no difference in the high K^+^-induced [Ca^2+^]_i_ rise between KO and WT cells (Fig. 5B). These data suggest that specific steps in glucose-induced early signal transduction that precede the [Ca^2+^]_i_ rise were impaired in KO cells.

### The responsiveness of the K_ATP_ channel current to glucose was impaired in *CDKAL1* KO β cells

**Figure 6:**
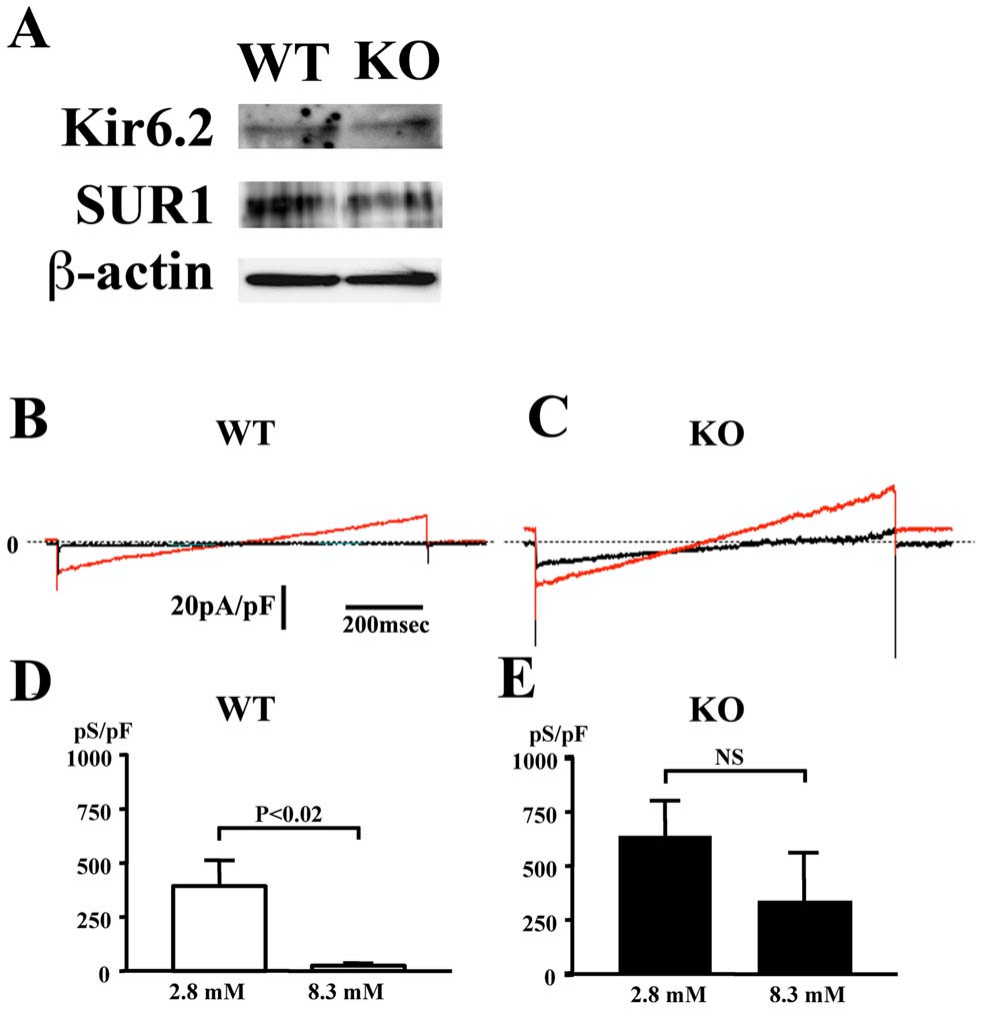
Effects of the increase in glucose concentration on K_ATP_ channel currents in WT and *CDKAL1* KO β-cells. **A**. Expression of Kir6.2 and SUR1 proteins in WT and KO islets by immunoblotting. Equal amounts of islet protein (30 µg) were separated by SDS-PAGE and immunoblotted. β-actin was used as a loading control. **B–E**. K_ATP_-channel currents expressed as current density in the ramp-clamp mode between −100 mV and −50 mV at a rate of 0.5 mV/ms in the presence of 2.8 mM (red line) or 8.3 mM glucose (black line) in WT (**B** and **D**) and KO β cells (**C** and **E**). Dotted lines indicate 0 A. Results are means±SEM and paired t-test was used to compare the data.

We next investigated the function of K_ATP_ channels in *CDKAL1* KO cells. As shown in Fig. 6A, there were no differences in the expression of the K_ATP_ channel components Kir6.2 and SUR1 between WT and KO islets. The K_ATP_ channel currents evoked by the voltage ramp from −100 to −50 mV in WT and KO mice are expressed as the current density determined as the calculated conductance (pS) divided by the cell capacitance (pF). The K_ATP_ currents were determined in the presence of 2.8 and 8.3 mM glucose in WT and KO mice (Fig. 6B and C). The current densities at 2.8 mM glucose were 392.9±121.6 pS/pF in WT (n = 6) and 639.5±145.1 pS/pF in KO cells (n = 8) (P>0.05). In WT cells, the K_ATP_ current density decreased significantly by increasing the glucose concentration from 2.8 to 8.3 mM (Fig. 6D; P<0.02), while there was a relatively small, non-significant decrease in the current density in KO mice (Fig. 6E; P>0.05). In addition, the time to the most inhibited level of K_ATP_ currents after exposing β cells to 8.3 mM glucose tended to be longer in KO cells (4.73±1.16 min, n = 5) compared with that in WT cells (3.11±0.48 min, n = 6; P>0.05). Thus our data indicate that the K_ATP_ channel responsiveness to glucose stimulation was blunted in *CDKAL1* KO β cells compared with WT cells.

On the other hand, we did not observe any differences in the ATP sensitivity of the channel in studies using inside-membrane patches. The half-maximal concentration of channel inhibition for ATP was 2.6 µM (n = 9) in *CDKAL1* KO mice (data not shown), which is less than those previously reported in control mice [13], [14]. These results suggest that changes in K_ATP_ channel activity in KO cells were not responsible for the reduced ATP sensitivity of these channels.

### ATP generation was impaired in KO cells

**Figure 7:**
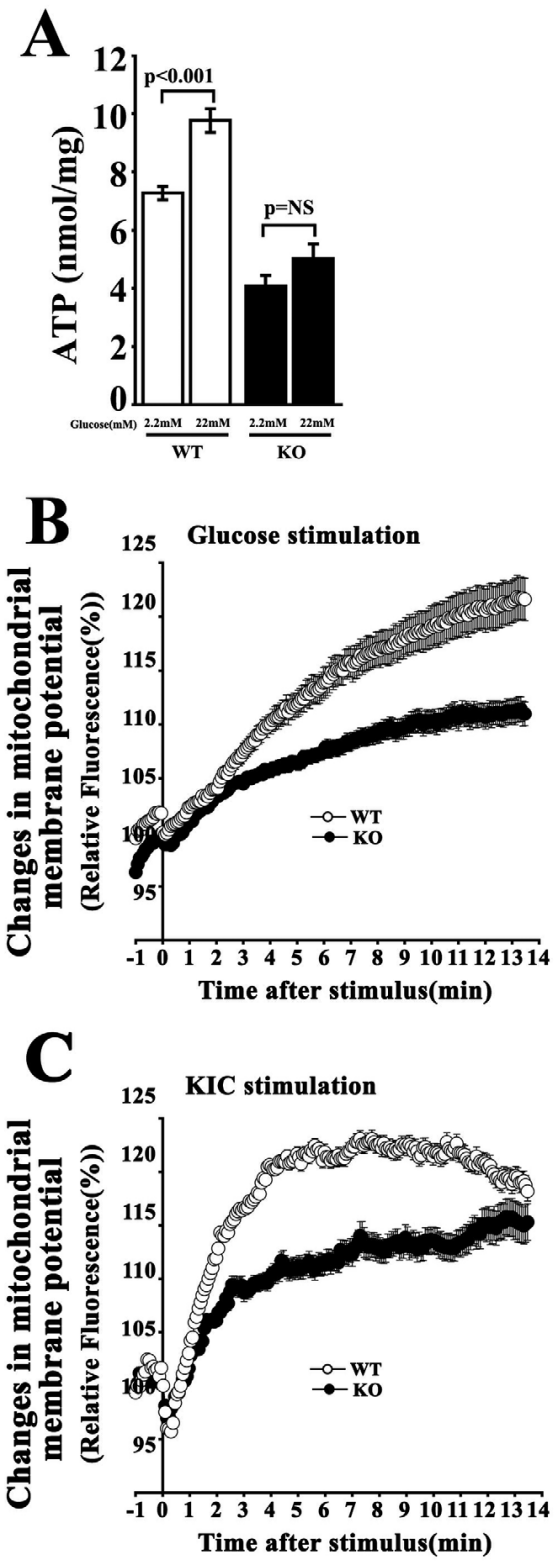
Effects of *CDKAL1* KO on ATP generation. **A**. ATP content in WT and *CDKAL1* KO islets. HPLC was used to measure ATP content in islets from WT and KO mice after incubation with 2 or 22 mM glucose. Results are means±SEM (n = 7 per group). **B**, **C**. Changes in mitochondrial membrane potential in response to 22 mM glucose (**B**) and 10 mM KIC (**C**) in WT and KO β cells. Changes in mitochondrial membrane potential were measured by a mitochondrial potential sensitive dye TMRE (10 nM). Time 0 indicates when the stimulants were added. The fluorescence intensity at time 0 was taken as 100%. Results are means±SEM (n = 12 cells per group).

It is conceivable that the reduced K_ATP_ channel responsiveness to glucose in KO cells might be due to a defect in ATP production following glucose metabolism. Therefore, we measured ATP content in islets from WT and KO mice after incubation with 2.2 and 22 mM glucose using high-performance liquid chromatography (HPLC) with a reverse-phase column. As shown in Fig. 7A, ATP content was significantly increased in WT islets by stimulation with 22 mM glucose (P<0.001; n = 7). In contrast, the ATP levels in the KO islets showed a small but not statistically significant increase, indicating impaired glucose metabolism in KO islets.

In line with glucose metabolism, we measured changes in the mitochondrial membrane potential in WT and KO cells during glucose stimulation using tetramethyl rhodamine ethyl ester (TMRE). Hyperpolarization of the mitochondrial membrane potential is evident by an increase in the fluorescent intensity of TMRE [15]. *CDKAL1* KO cells exhibited a significantly reduced membrane potential response to glucose stimulation compared with WT cells (Fig. 7B). The reduced mitochondrial membrane potential response was further confirmed using α-ketoisocaproate (KIC) (Fig. 7C), which is a direct substrate for the mitochondrial TCA cycle and bypasses glycolysis [16]. Thus, it seems reasonable to conclude that the defective ATP generation in KO cells is due to impaired mitochondrial function, without changes in mitochondrial number or morphology in KO cells on electron microscopy (data not shown).

### CDK5/p35 protein kinase activity in *CDKAL1* KO islets

**Figure 8:**
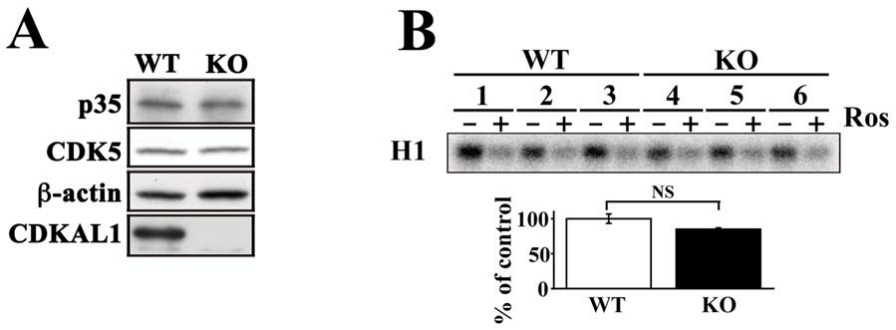
CDK5/p35 protein kinase activity in *CDKAL1* KO islets. **A**. Detection of CDK5 and p35 proteins in WT and KO islets by immunoblotting. Equal amounts of islet protein (20 µg) were separated by SDS-PAGE and immunoblotted. β-actin was used as a loading control. **B**. Detection of CDK5/p35 kinase activity in WT (lanes 1–3) and KO (lanes 4–6) islets. After immunoprecipitation with a p35-specific antibody in WT and KO islets, kinase activity was measured using histone H1 as the substrate in the presence (+) or absence (−) of a CDK5 inhibitor roscovitine (Ros; 20 µM). Quantification is shown in the lower panel as means±SEM (n = 3 per group).

CDKAL1 is homologous to CDK5RAP1, which inhibits CDK5 activity by binding to the kinase activator p35 [6]. To test whether CDK5-mediated regulation is involved in the function of CDKAL1 in β cells, we examined the expression and protein kinase activity of CDK5 in *CDKAL1* KO islets. CDK5 and p35 were expressed in KO islets as much as in WT islets (Fig. 8A). Surprisingly, we found no difference in CDK5/p35 kinase activity between *CDKAL1* KO and WT islets, when using histone H1 as a substrate after immunoprecipitation with a p35-specific antibody (Fig. 8B). The specificity of this activity was confirmed using the CDK5 inhibitor roscovitine. Thus, it seems likely that CDK5 kinase does not contribute to CDKAL1 function in β cells.

### Subcellular localization of CDKAL1 in β cells

**Figure 9:**
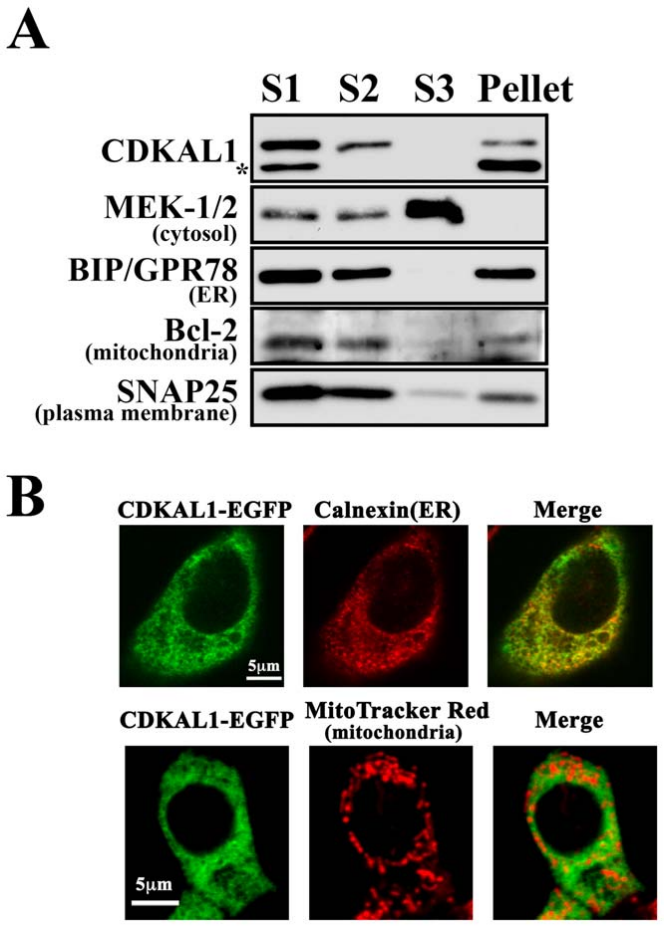
Subcellular localization of CDKAL1 in β cells. **A**. Subcellular fractionation for CDKAL1 localization. The postnuclear fraction (S1) from MIN6 β cells homogenates was fractionated by differential centrifugation (see Methods). Equal amounts (2 µg protein) of the fractions obtained were analyzed by immunoblotting with the indicated antibodies. S2, soluble cytosolic/microsomal fraction; Pellet, membrane fraction; S3, soluble cytosolic fraction. The protein band below the CDKAL1 protein (*) is a nonspecific protein band detected by the anti-CDKAL1 antibody. **B**. Immunocytochemical localization of CDKAL1 in MIN6 β cells. MIN6 β cells were transfected with EGFP–CDKAL1 and intracellular localization was examined using a confocal microscope with MitoTracker Red staining or immunostaining with anti-calnexin IgG (ER marker), as indicated.

Finally, we examined the subcellular localization of CDKAL1 using a biochemical subcellular fractionation of MIN6 β cells. In the fractionation procedure [17], the postnuclear S1 fraction was separated by differential centrifugation into an S2 soluble cytosolic/microsomal fraction and a pellet membrane fraction. Then, the S2 fraction was separated into the S3 soluble cytosolic fraction. Endogenous proteins were used as markers for cytosolic (MEK-1/2), endoplasmic reticulum (ER) (BIP/GRP78), mitochondrial (Bcl2), and plasma membrane proteins (SNAP25). CDKAL1 immunoreactivities were detected in the S2 and pellet fractions, but not in the S3 soluble cytosolic fraction (Fig. 9A), suggesting that CDKAL1 seems to associate with microsomes or plasma membrane.

We next performed immunocytochemistry to confirm the subcellular localization of CDKAL1 in β cells. The CDKAL1 antibody cross-reacted with a non-specific protein band (Fig. 1B and Fig.9A), suggesting that the antibody is not suitable for immunostaining. In fact, in immunostaining experiments with the CDKAL1 antibody, we could not detect any specific signal in WT cells (data not shown). Therefore, we expressed the CDKAL1 protein as a fusion with enhanced green fluorescent protein (EGFP) and the intracellular localization was examined under a confocal microscope immunostaining with organelle markers or with MitoTracker staining. As shown in Fig. 9B, the fluorescence of the CDKAL1–EGFP protein was colocalized with the ER protein calnexin, but not with MitoTracker Red (a mitochondria marker). These results suggest that CDKAL1 is predominantly localized to the ER.

## Discussion

In this study, we found that pancreatic β cells from mice null for *CDKAL1*, which was identified as a susceptibility gene for type 2 diabetes in humans, have a reduced first-phase insulin release *in vitro*. TIRF imaging revealed that *CDKAL1* KO caused a marked reduction in the number of fusion events during first-phase release (Fig. 3), but not in second-phase release. The impaired glucose-induced first-phase insulin exocytosis was associated with a delayed and slow increase in [Ca^2+^]_i_ during glucose stimulation in KO cells (Fig. 5). This may be due to the blunted responsiveness of K_ATP_ channels to glucose stimulation in KO cells (Fig. 6). Indeed, ATP production in KO cells was low (Fig. 7) and may lead to blunted glucose-induced K_ATP_ channel responsiveness followed by impaired Ca^2+^ channel activity.

The target molecules for CDKAL1 are not yet known, although our data indicate that *CDKAL1* KO influences glucose-induced K_ATP_ channel responsiveness through reduced ATP generation. Since CDKAL1 is similar to CDK5RAP1, we originally speculated that the function of CDKAL1 is mediated by CDK5, and is probably directly associated with increased Ca^2+^ channel activity, as observed in p35 KO mice [8], or with insulin gene expression [7], [9]. However, there was no relationship between CDKAL1 and CDK5 in this study. Therefore, CDK5/p35-mediated regulation does not seem to be involved in CDKAL1 regulation of insulin release. Our data suggest that CDKAL1 is instead related to glucose metabolism, which is associated with ATP generation, K_ATP_ channel activity and Ca^2+^ channels activity.

The failure of glucose-induced ATP-generation may be due to defects at several stages of glucose metabolism including cytosolic glycolysis and mitochondrial metabolism. In *CDKAL1* KO cells, the glucose-stimulated mitochondrial membrane potential response was blunted (Fig. 7B). In addition, KIC, which is a direct substrate for the TCA cycle, bypassing glycolysis, also reduced the mitochondrial membrane potential response (Fig. 7C). Therefore, blunted mitochondrial membrane potential response is likely to be a key defect, resulting in a reduced ATP generation, K_ATP_ channel responsiveness and Ca^2+^ channel activity, and eventually leading to impaired insulin granule exocytosis in KO cells. Indeed, it has been reported that the selective impairment of glucose-induced insulin release in diabetic Goto-Kakizaki rats is associated with reduced sensitivity of K_ATP_ channels to glucose by deficient oxidative metabolism of glucose in mitochondria [18], [19].

In KO cells, cytosolic free [Ca^2+^]_i_ induced by glucose gradually reached the same level observed in WT cells. We assume that the increase of [Ca^2+^]_i_ during second-phase may reflect suppression of Ca^2+^ clearance mechanism. As for other animal cells, four mechanisms remove Ca^2+^ from cytosol of β cells: sarco-endoplasmic reticulum Ca^2+^-ATPase (SERCA) pumps, plasma membrane Ca^2+^-ATPase (PMCA), Na^+^/Ca^2+^ exchanger (NCX), and the calcium uniporter of mitochondria [20], [21]. In mouse and rat β cells, the SERCA pump is the dominant Ca^2+^ clearance mechanism [20], [21], thus it is conceivable that reduced ATP generation impairs the Ca^2+^ clearance function via the SERCA pump, resulting that there is no difference of [Ca^2+^]_i_ in second-phase between KO and WT cells.

As shown in Fig. 9, CDKAL1 is likely to localize to the ER in β cells. The relationship between CDKAL1 deletion in the ER and reduced mitochondrial ATP generation remains unknown. However, it has been reported that the ER and mitochondria are in close contact, which supports communication between these two organelles [22]. It is possible that ER dysfunction caused by *CDKAL1* KO may affect mitochondrial function in the KO cells. Such ER–mitochondria communication has been shown to involve the ER stress pathway [22], [23], even in β cells [24], [25]. Very recently, Arragain et al. reported that CDKAL1 is a methylthiotransferase involved in the biosynthesis of 2-methythio-N6-threonylcarbamoyladenosine in transfer RNA (tRNA) [26]. The methylthio modification of adenosine in tRNA appears to be essential for efficient and highly accurate protein translation by the ER ribosome. However, it is still unclear how the role of CDKAL1 in tRNA may be linked to ER function or ATP generation in mitochondria. Thus, it is tempting to speculate that either ER dysfunction or ER stress may affect the mitochondrial membrane potential in KO cells.

It is particularly interesting to note that our TIRF imaging data showed that CDKAL1 deletion only impaired the first-phase, but not the second-phase, of glucose-induced insulin exocytosis. Our data are in good agreement with the results of Groenewoud et al. [10] and Stancakova et al. [11], who reported that *CDKAL1* variants decreased first-phase insulin secretion but not second-phase insulin in humans with type 2 diabetes. Thus, the altered expression of CDKAL1 is probably associated with reduced glucose-induced first-phase insulin exocytosis and thus confers an increased risk for diabetes.

Although our data showed a critical role of CDKAL1 in insulin exocytosis *in vitro*, the *CDKAL1* KO mice had almost normal glucose homeostasis (N. Kato, in submission). The reason for the normal glucose homeostasis *in vivo*, despite reduced first-phase insulin release in vitro, is unknown, but there are several possibilities to be considered. First, the magnitude of the reduction in first-phase insulin release is not sufficient to cause impaired glucose tolerance in mice at this stage we examined. In fact, Stancakova et al. [11] reported that the SNP rs7754840 of *CDKAL1* is associated with impaired insulin secretion in non-diabetic offspring of type 2 diabetic subjects and in a large sample of men with normal glucose tolerance. Second, the role of CDKAL1 on the regulation of insulin exocytosis may have a limited influence on whole-body glucose metabolism. Therefore, additional genetic and environmental factors may be needed to cause impaired glucose tolerance.

In summary, we have provided the first report documenting a role of CDKAL1 in β cells. Our study using *CDKAL1* KO mice shows that CDKAL1 controls first-phase insulin exocytosis in β cells by facilitating ATP generation, glucose-induced K_ATP_ channel responsiveness and subsequent Ca^2+^ channel activity. Defects in this process caused by decreased CDKAL1 expression levels may confer an increased risk of diabetes.

## Methods

### Generation of *CDKAL1* KO mice

*CDKAL1* KO mice were generated by the gene-trapping method [27]–[29] at TransGenic Inc. (Kobe, Japan) and thereafter established as an experimental model at the National Center for Global Health and Medicine. Male mice were used at the age of 10–14 weeks. Animal experiments were approved by the Animal Care and Use Committee of NCGM Research Institute (permit number: 22-TG-35) and the Kyorin University Animal Care Committee (permit number: 65-2).

### Islets and pancreatic β cells

Pancreatic islets of Langerhans were isolated from male WT and *CDKAL1* KO mice by collagenase digestion, as previously described [30]. The isolated islets were then dispersed and cultured [30]. To label the insulin secretory granules, pancreatic β cells were infected with recombinant adenovirus Adex1CA insulin–GFP [30].

### RT-PCR

Total RNA was extracted using an Illusta kit (GE healthcare, Piscataway, NJ, USA), and the full-length *CDKAL1* was amplified by RT-PCR using specific primers (forward primer: ctctcagttcggacagattcatctttcaagaggac; reverse primer: gctgtgatctggtctgaggtcagatggc; product 2 kb). The PCR product was separated on agarose gels.

### Immunoblotting

Proteins were extracted from mouse pancreatic islets and whole brain and immunoblotted [12], [31]. We used antibodies targeting the following proteins: CDKAL1 (ab68045, Abcam, Cambridge, UK), p35 (C19, Santa Cruz, CA, USA), CDK5 (DC17, Santa Cruz), syntaxin 1A (a gift from Dr. T. Fujiwara, Kyorin University School of Medicine, Tokyo, Japan), SNAP25 (Wako Pure Chemical Industries Ltd., Osaka, Japan), VAMP2 (StressGen Biotechnologies Corp, Victoria, BC, Canada), Kir6.2 (Santa Cruz), SUR1 (Santa Cruz), MEK-1/2 (Cell Signaling Technology, Beverly, MA, USA), BIP/GPR78 (BD Transduction Laboratories, Lexington, KY, USA), Bcl-2 (BD Transduction Laboratories) and β-actin (Sigma-Aldrich, St. Louis, MO, USA).

### Morphometric analysis of islets

To analyze islet size and β cell mass, paraffin-embedded pancreas sections (10 µm) were labeled with anti-insulin antibodies and detected by an avidin-biotin-peroxidase technique (Vector Laboratories, Burlingame, CA, USA). Sections were collected at 500 µm intervals from tissue blocks, and all islets in the sections were analyzed to determine islet area as a percentage of total pancreatic area. Images were acquired with an Olympus microscope IX70 equipped with a charge-coupled device camera, and analyzed with Metamorph software (Molecular Devices, Downingtown, PA, USA).

### Electron microscopy

Electron microscopy was carried out using previously described methods [12]. Tissues were fixed in phosphate-buffered 2.5% glutaraldehyde (pH 7.4), postosmicated, dehydrated with graded alcohols, and embedded in Epon 812. After staining with uranyl acetate and lead citrate, ultra-thin sections were examined under a transmission electron microscope (TEM-1010C, JEOL, Akishima, Tokyo, Japan). In electron microscopy, granules at their shortest distance of <10 nm from the plasma membrane were defined as morphologically docked granules.

### Insulin content

To measure insulin content in the whole pancreas, the excised pancreata were frozen in liquid nitrogen and disrupted with Cryopress (Microtech Nichion, Funabashi, Japan). The resulting powder was then suspended in cold acid-ethanol, and insulin was extracted overnight at 4°C [32]. The supernatants were diluted and subjected to enzyme-linked immunosorbent assays (ELISA) as described previously [12]. Insulin content per isolated islet was measured by ELISA [12]. Hoechst-33258 staining of sonicated islets was performed to determine the islet DNA content.

### Insulin release in batch incubated β cells

Batch incubation experiments for insulin release have been described elsewhere [33]. Briefly, after plating WT and KO cells on 96-well plates, the cells were preincubated for 30 min in Krebs-Ringer buffer (KRB) containing (in mM): 110 NaCl, 4.4 KCl, 1.45 KH_2_PO_4_, 1.2 MgSO_4_, 2.3 calcium gluconate, 4.8 NaHCO_3_, 2.2 glucose, 10 HEPES, pH 7.4, and 0.3% bovine serum albumin (BSA), and then challenged with 2.2 mM glucose or 22 mM glucose for 30 min. At the end of the stimulation period, the cells were disrupted by sonication and aliquots of media and cell extracts were analyzed by ELISA. The released insulin is expressed as a percentage of total cellular content.

### TIRF microscopy

The Olympus total internal reflection system with a high-aperture objective lens was used as described previously [12]. Briefly, primary β cells expressing insulin–GFP on the glass cover slip (Olympus, Tokyo, Japan) were mounted in an open chamber and incubated for 30 min with KRB. Cells were then transferred to a thermostat-controlled stage (37°C) and stimulated with 22 mM glucose (final) and 40 mM KCl (final). To evaluate the number of docked insulin granules by TIRF microscopy, wild-type and *CDKAL1* knockout cells were cultured on high refractive index glass, fixed, permeabilized with 4% paraformaldehyde/0.1% Triton X-100, and processed for immunohistochemistry [12]. Cells were labeled with anti-insulin antibodies (Sigma-Aldrich) and processed with goat anti-mouse IgG conjugated to Alexa Fluor-488 (Molecular probes, Eugene, OR, USA). Immunofluorescence was detected by TIRF microscopy.

### Measurements of intracellular free Ca^2+^ ([Ca^2+^]_i_)

Fura-2 acetoxymethyl ester (Fura-2 AM: Molecular Probes) was loaded into cultured β cells and the ARGUS/HiSCA system (Hamamatsu photonics, Hamatsu, Japan) was used for measurement, as previously described [12].

### Patch-clamp experiments in mouse single β cells

Perforated whole-cell currents were recorded using a pipette solution containing amphotericin B (200 µg/ml) dissolved in 0.1% DMSO [34]. Membrane currents were recorded using an amplifier (200B; Axopatch, Foster, CA) and stored online in a computer running pCLAMP10.2 software. The voltage clamp in perforated mode was considered to be adequate when the series resistance was <20 MΩ. Patch pipettes were pulled from glass tubes (Narishige, Tokyo, Japan); the resistances of the pipettes ranged from 4 to 7 MΩ when filled with the pipette solution containing 40 mM K_2_SO_4_, 50 mM KCl, 5 mM MgCl_2_, 0.5 mM EGTA and 10 mM HEPES at pH 7.2 with KOH. To record the K_ATP_ channel current, the β cells were voltage-clamped to the holding potential of −70 mV, stepped to −100 mV to apply the voltage ramp from −100 to −50 mV at a speed of 5 mV/100 ms, and stepped back to −70 mV every 10 s. Electrophysiological experiments were performed at room temperature (25°C).

### Measurement of islet ATP content

The ATP content of pancreatic islets was measured by HPLC using a modified version of previously described methods [35], [36]. Experiments were performed on islets from WT and KO mice after incubation overnight in RPMI containing 11 mM glucose. Groups of 25 islets were hand-picked and preincubated in KRB containing 2.2 mM glucose for 60 min. After preincubation, the islets were incubated for 5 min in KRB containing 2.2 or 22 mM glucose. Then, ATP was extracted with 0.4 M perchloric acid and assayed by HPLC with a reversed-phase SUPELCOSIL column (LC-18-T, 3 µm, 15×4.6 mm, Sigma-Aldrich) according to the manufacturer's instructions. ATP levels were standardized to protein concentrations.

### Mitochondrial membrane potential

WT and KO cells cultured on cover glass were loaded with 10 nM TMRE (Molecular Probes) [15] for 45 min in KRB containing 2.2 mM glucose and washed and incubated for a further 15 min with KRB. We obtained TMRE fluorescence images from multiple individual cells before and after increasing the extracellular glucose concentration from 2.2 mM to 22 mM or with 10 mM KIC (Nakarai Tesque Inc, Kyoto Japan) using confocal fluorescent microscopy (FV-1000, Olympus) [543-nm laser line and emission filter set (BA 560–620)].

### Immunoprecipitation and detection of the histone H1 kinase activity of CDK5

The kinase activity of CDK5/p35 was measured after immunoprecipitation with anti-p35 antibody, as previously described [31]. Briefly, the isolated islets were sonicated in MOPS buffer (20 mM MOPS, pH 6.8, 1 mM EGTA, 0.1 mM EDTA, 0.3 M NaCl, 1 mM MgCl_2_, 0.5% Nonidet P-40, 0.2 mM Pefabloc SC, 1 mg/ml leupeptin, and 1 mM DTT), and centrifuged at 20,000×g for 30 min to collect the supernatant. The kinase activity of CDK5-p35 immunoprecipitated with anti-p35 antibody (C19, Santa Cruz) from an equal protein amount of islet extract was measured using histone H1 as a substrate in the absence or presence of 20 µM roscovitine.

### Subcellular localization of CDKAL1 in β cells

Fractionation by differential centrifugation was performed with minor modification as described previously [17]. MIN6 β cells (a gift from Dr. J.-I. Miyazaki, Osaka University, Osaka, Japan) were homogenized in sucrose buffer (5 mM HEPES (pH 7.5), 0.32 M sucrose, and protease inhibitors). The homogenates were centrifuged at 800×g for 10 min to obtain the postnuclear supernatant (S1). S1 was centrifuged at 8,900×g for 15 min to separate the S2 soluble cytosolic/microsomal fraction and precipitate. The precipitate was resuspended in sucrose buffer, and centrifuged at 540,000×g for 2 h, to yield the pellet (membrane fraction). The S2 fraction was centrifuged for 1 h at 540,000×g for 2 h, yielding a soluble cytosolic fraction (S3). Equal amounts (2 µg protein) of the fractions were separated by SDS-PAGE and immunoblotted with specific antibodies.

EGFP–CDKAL1 was generated using a cDNA fragment encoding a full open reading frame of murine CDKAL1 (Dnaform, Kanagawa, Japan), N-terminally tagged with EGFP, and subcloned into the HindIII/KpnI site of a pcDNA3 vector (Invitrogen). For immunohistochemical localization of CDKAL1, MIN6 β cells were transfected with EGFP–CDKAL1 using Lipofectamine2000 (Invitrogen) according to the manufacturer's instructions and cultured for 2 days. The intracellular localization was examined under a confocal microscope with MitoTracker (Red CM-H2XRos, Molecular probes) staining (100 nM for 20 min) or immunostaining (fixed and permeabilized with 4% paraformaldehyde/0.1% Triton X-100) with anti-calnexin antibody.
